# Experiences and perceived outcomes of a grocery gift card programme for households at risk of food insecurity

**DOI:** 10.1017/S136898002300157X

**Published:** 2023-11

**Authors:** Yun Yun Lee, Stéphanie Caron-Roy, Bobbi Turko, Jane Shearer, David JT Campbell, Charlene Elliott, Donald Barker, Kim D Raine, Sheila Tyminski, Dana Lee Olstad

**Affiliations:** 1 Faculty of Kinesiology, University of Calgary, 2500 University Drive NW, Calgary, AB T2N 1N4, Canada; 2 I Can for Kids, 26 Riverview Park SE, Calgary, AB T2C 3Z7, Canada; 3 Department of Community Health Sciences, Cumming School of Medicine, University of Calgary, 3280 Hospital Drive NW Calgary, AB T2N 4Z6, Canada; 4 Department of Communication, Media and Film, University of Calgary, 2500 University Drive NW, Calgary, AB T2N 1N4, Canada; 5 School of Public Health, University of Alberta, 3-300 Edmonton Clinic Health Academy, 11405-87 Ave Edmonton, AB T6G 1C9, Canada; 6 Nutrition Services, Alberta Health Services, Calgary, AB, Canada

**Keywords:** Household food insecurity, food subsidy programme, food assistance, child nutrition, qualitative description

## Abstract

**Objective:**

This study explored programme recipients’ and deliverers’ experiences and perceived outcomes of accessing or facilitating a grocery gift card (GGC) programme from I Can for Kids (iCAN), a community-based programme that provides GGC to low-income families with children.

**Design:**

This qualitative descriptive study used Freedman et al’s framework of nutritious food access to guide data generation and analysis. Semi-structured interviews were conducted between August and November 2020. Data were analysed using directed content analysis with a deductive–inductive approach.

**Participants:**

Fifty-four participants were purposively recruited, including thirty-seven programme recipients who accessed iCAN’s GGC programme and seventeen programme deliverers who facilitated it.

**Setting:**

Calgary, Alberta, Canada.

**Results:**

Three themes were generated from the data. First, iCAN’s GGC programme promoted a sense of autonomy and dignity among programme recipients as they appreciated receiving financial support, the flexibility and convenience of using GGC, and the freedom to select foods they desired. Recipients perceived these benefits improved their social and emotional well-being. Second, recipients reported that the use of GGC improved their households’ dietary patterns and food skills. Third, both participant groups identified programmatic strengths and limitations.

**Conclusion:**

Programme recipients reported that iCAN’s GGC programme provided them with dignified access to nutritious food and improved their households’ finances, dietary patterns, and social and emotional well-being. Increasing the number of GGC provided to households on each occasion, establishing clear and consistent criteria for distributing GGC to recipients, and increasing potential donors’ awareness of iCAN’s GGC programme may augment the amount of support iCAN could provide to households.

Household food insecurity refers to inadequate or insecure access to food due to financial constraints^(1)^. Inadequate income is a key determinant of household food insecurity^(2)^. According to the most recent nationally representative data from 2021, 15·9 % of households in Canada were food-insecure, including 19·6 % of children who lived in food-insecure households^(3)^. Amongst the provinces, Alberta had the highest rate of food insecurity at 20·3 %, including 21·7 % of children who lived in food-insecure households^(3)^.

Among food-insecure households with children, food insecurity has been associated with poor diet quality^(4–6)^, physical health^(7–9)^, mental well-being^(10–13)^ and academic performance^(14–17)^ among children. Food subsidy programmes, such as the Supplemental Nutrition Assistance Program (SNAP) in the USA and grocery gift card (GGC) programmes in several nations may be an important means to reduce household food insecurity by providing funds to purchase adequate, nutritious foods for all household members^(18,19)^. Quantitative evidence suggests that food subsidy programmes can reduce the severity of household food insecurity and improve the diet quality of children and other household members^(19–21)^. Several qualitative studies have explored the experiences of participating in food subsidy programmes and found that recipients perceived financial, dietary and social benefits, including the autonomy to select and procure foods that met their households’ food preferences^(22,23)^. However, recipients still perceived that the monetary value of food subsidies and the frequency with which they were provided was inadequate^(22,23)^. A small number of qualitative studies have also explored programme deliverers’ experiences and perceived outcomes of facilitating food subsidy programmes. Programme deliverers from these studies described that participants’ access to food within food subsidy programmes was constrained by administrative and operational aspects of programme delivery, including limited funding and personnel, and complex enrolment processes^(24,25)^.

The number of GGC programmes in Canada has increased to address an increased prevalence of food insecurity with ongoing economic instability due to the COVID-19 pandemic^(26–28)^. However, qualitative studies have not yet explored the experiences and perceived outcomes of individuals accessing or delivering GGC programmes. As such, it is not known whether and how GGC can support food access among households at risk of food insecurity.

## Programme overview and objectives

I Can For Kids (iCAN) is an organisation that implemented a GGC programme in Calgary, Alberta, Canada (a city of approximately 1·3 million people^(29)^) in response to the COVID-19 pandemic. iCAN is a non-profit organisation that was started in 2015 and initially distributed nutritious food packs to school-aged children from low-income households in the summer months when they no longer had access to school meals^(30)^. In April 2020, iCAN transitioned from a summer food pack programme to a year-round GGC programme^(31)^. The aim of providing GGC was to address the immediate food needs of children under 18 years of age living in low-income households who struggled to access food due to the economic impacts of the COVID-19 pandemic^(31)^.

iCAN raises funds to operate its GGC programme from corporate and private donors. As such, the availability of funds to purchase GGC fluctuates from year to year. iCAN purchases GGC from almost every major grocery retail chain in Calgary. Each GGC is valued at CAD $50 and can be used to purchase any item at any food store affiliated with the grocery retail chain that issued the GGC. iCAN does not distribute GGC themselves, but instead have built partnerships with community agencies in the city that serve households with children under 18 years of age (e.g. community resource centres and subsidised family housing complexes) to distribute GGC. Agencies request GGC for grocery stores they believe their clients are likely to shop at (e.g. stores that are nearby or with lower prices), and recipients can select from among the GGC that agencies requested. Agencies also determine the number of GGC to provide to each household and how often (e.g. bi-weekly or monthly) on a case-by-case basis (i.e. depending on each agency’s allotment of GGC from iCAN and household food needs). For example, most households receive GGC valued at CAD $50 per visit; however, larger households often receive GGC valued at CAD $100 per visit.

Through the ongoing support of and partnerships with funders and community agencies, iCAN reached 136 communities, supporting over 28 000 children with GGC valued at more than CAD $714 000 between April and December 2020^(31)^. As iCAN’s GGC programme continues to expand and provide GGC to more households at risk of food insecurity, it is imperative to explore whether and how accessing GGC reduces the risk of household food insecurity and enhances children’s access to sufficient, nutritious food. Therefore, the aim of this study was to explore programme recipients’ and deliverers’ experiences and perceived outcomes of accessing or facilitating iCAN’s GGC programme.

## Methods

### Methodology and theoretical framework

This qualitative descriptive study aimed to generate rich descriptions of participants’ experiences and perceived outcomes of accessing or facilitating iCAN’s GGC programme^(32)^. Data generation and analysis were guided by Freedman et al’s^(33)^ theoretical framework of nutritious food access. This framework posits that food access is shaped by a comprehensive range of factors within five key domains: economic (e.g. household financial resources), service delivery (e.g. access to fresh, unexpired food), spatial-temporal (e.g. travel distance to grocery stores), social (e.g. cultural food preferences) and personal (e.g. health-related food needs)^(33)^. Given that little is known about GGC programmes, the comprehensive nature of Freedman et al’s^(33)^ framework may help to gain in-depth insight into what may have facilitated or constrained recipients’ access to food via iCAN’s GGC programme.

### Recruitment and data generation

Participants were purposively recruited in collaboration with the iCAN Director and nine community agencies throughout Calgary in an attempt to enrol a diverse group of recipients who differed in household size, household type and immigrant status. Programme recipients were recruited in collaboration with community agency staff, whereby staff approached recipients about the study via face-to-face discussion or by phone. To be eligible for the study, programme recipients had to be a primary caregiver of the children in the household, received and used at least two GGC from iCAN in the current programme year (2020), accessed food support from iCAN or other food support programmes in previous programme years, the primary food shopper and food preparer in the household, and be able to speak and understand English.

Programme deliverers were also purposively recruited in an attempt to recruit deliverers who served different populations (e.g. tenants of subsidised housing and residents of low-income neighbourhoods). Programme deliverers from community agencies were recruited by Y.Y.L. via face-to-face discussion or by email. Programme deliverers were community agency staff involved in planning the GGC distribution process and/or providing GGC to programme recipients, but who did not work for iCAN. Programme deliverers were eligible for the study if they had facilitated iCAN’s programme for at least 2 years, provided GGC to at least two households during the current programme year (2020) and provided food from iCAN or connected recipients to other food support programmes in previous programme years. Sampling for both participant groups continued until saturation was reached. Saturation was based on the code meaning approach, whereby no new codes, or meanings of codes, were being identified in the data^(34)^.

Data generation occurred between August and November 2020. Two semi-structured interview guides (see online Supplementary Materials) were developed using Freedman et al’s^(33)^ framework that were specific to each participant group and were pretested with three programme recipients and one programme deliverer. These data were included in the analyses as they yielded valuable descriptions of participants’ experiences and perceived outcomes of accessing or delivering iCAN’s GGC programme. A total of fifty-four interviews were conducted with thirty-seven programme recipients and seventeen programme deliverers in-person, by phone, or virtually by Y.Y.L. and S.C.R. Written or verbal informed consent was obtained from all participants prior to interviews. Interviews were approximately 45–80 min in length. All participants received CAD $25 cash for participating. All interviews were audio-recorded and transcribed verbatim, with the exception of one where the participant declined to be audio-recorded. For the interview that was not recorded, detailed notes of the participant’s responses were manually recorded by the researcher conducting the interview. As data generation and analysis occurred concurrently, the interview guide was continuously adapted to better capture emerging concepts.

### Data analysis

Data analysis was an iterative and interactive process as concurrent data generation and analysis mutually shaped one another. Directed content analysis was used to analyse the data, whereby Freedman et al’s^(33)^ framework informed the initial coding schemes. Analysis began with repeated listening to the audio recordings and reading of transcripts. Codebooks were developed by two researchers (Y.Y.L. and S.C.R.). The researchers coded the first three programme recipient interviews independently using the initial coding scheme. Data that did not fit with the initial coding scheme were coded inductively. The researchers met to review and discuss different perspectives of the coded data. The coding scheme was then revised to enhance the reliability of the coding process. Researchers applied the revised coding scheme to two more recipient interviews independently after which they met to review and discuss the coded data and finalised the coding scheme. The same process was used to develop and refine a coding scheme for programme deliverer interviews. Once both codebooks were finalised, Y.Y.L. coded the remaining interviews. Y.Y.L. then collated and organised codes to form subthemes and themes, which were further developed and refined after discussion with the research team.

Strategies to enhance rigour included maintaining reflexive notes, recording field notes after each interview and keeping a detailed audit trail throughout the research process. Investigator triangulation, peer debriefing throughout data generation and analysis and presenting verbatim quotes from participants alongside study findings helped to establish descriptive validity. Pseudonyms have been used for verbatim quotes.

## Results

Programme recipient and programme deliverer characteristics are presented in Table 1. Just over half of programme recipients were immigrants to Canada (51 %) and 79 % of recipients were between the ages of 18–45 years. Participants reported a range of educational attainments, including post-secondary or higher (32 %), less than high school (24 %), or a high school diploma (22 %). One-half lived in a two-parent household and had 1–2 children living in the home. Most participants were from households at risk of food insecurity (84 %). The majority of programme deliverers were frontline staff (71 %) from community organisations that had partnered with iCAN for 2–3 years (76%).

**Table 1:** Characteristics of grocery gift card programme recipients and deliverers

Programme recipient characteristics (*n* 37)	Born in Canada		Immigrant		Total	
	*n*	%*	*n*	%*	*n*	%*
	18	49	19	51	37	100
Sex						
Female	17	94	10	53	27	73
Male	1	6	9	47	10	27
Age (years)						
18–35	9	50	5	26	14	38
36–45	8	44	7	37	15	41
46–55	0	0	5	26	5	14
56+	1	6	2	11	3	8
Educational attainment						
Less than high school	4	22	5	26	9	24
High school diploma	3	17	5	26	8	22
Trade certificate	2	11	0	0	2	5
Some post-secondary	4	22	1	5	5	14
Post-secondary or higher†	4	22	8	42	12	32
Don’t know/prefer not to answer	1	6	0	0	1	3
Household type						
Single-parent	13	72	5	26	18	49
Two-parent	5	28	14	74	19	51
Number of children living in the household						
1–2	10	56	9	47	19	51
3–6	8	44	10	53	18	49
At risk of household food insecurity‡						
Yes	15	83	16	84	31	84
No	3	17	3	16	6	16
GGC received§						
< 10 GGC	12	67	14	74	26	70
10–20 GGC	3	17	1	5	4	11
21–30 GGC	1	6	2	11	3	8
> 30 GGC	1	6	0	0	1	3
Don’t know	1	6	2	11	3	8

Programme deliverer characteristics (*n* 17)	Total *n*				%*	
Position at agency						
Frontline staff	12				71	
Manager	4				24	
Director	1				6	
Duration of iCAN partnership						
2–3 years	13				76	
4+ years	4				24	

GGC, grocery gift cards; iCAN, I Can for Kids.

* Percentages may not add up to 100 % due to rounding.

† Five immigrant recipients completed post-secondary degrees outside Canada.

‡ Based on Hagar et al’s two-item screener to identify households at risk of food insecurity. Experiences were reported for the 3 months prior to the interview.

§ Based on when recipients started receiving GGC until the time of their interview (between 4 and 7 months).

Three themes and corresponding subthemes were generated from the data to describe participants’ experiences of participating in and facilitating iCAN’s GGC programme. The first theme described how iCAN’s GGC programme promoted a sense of autonomy and dignity among programme recipients as they described experiencing financial support, the flexibility and convenience of using GGC, and the freedom to select foods they desired. Recipients reported that these benefits led to improved social and mental well-being. The second theme was related to how the use of GGC improved households’ dietary patterns and food skills. In the third theme, programme recipients and deliverers identified programmatic strengths and limitations.

### Theme 1: Autonomy and dignity

#### Competing household expenses

Recipients reported that GGC supplemented their household food budgets, which allowed them to afford other basic expenses, such as rent and utilities. Participants indicated that having fewer competing household expenses improved their emotional well-being as they felt less stressed over their household finances and food supply. Recipients perceived that when they felt less stressed, their children also experienced lower levels of stress. Recipients also described being less likely to access food banks and community agency food pantries for food and were less likely to borrow money from family members to buy food. Some recipients stated that with the support of GGC to afford food, they were able to reallocate their income to save for an emergency fund, which allowed them to achieve greater financial self-sufficiency. As Anna (two-parent household, four children) described, *‘[Having GGC] makes me happy because part of living [in subsidized housing] is trying to get out of here, to be self-sustainable… [GGC] gives me an opportunity to put money away and save for the things that we need’*. With fewer competing household expenses, some recipients were also able to set aside funds to provide ‘special moments’ for their children that they perceived further enhanced their ability to provide for their children. These moments included celebrating children’s birthdays or having picnics in the park. For instance, Hilary (two-parent household, four children) described feeling relief and joy at being able to provide her children ‘special moments’ that were previously not possible because of household financial constraints: *‘…all my kids have heard is ‘No’: ‘No you can’t do this’. ‘No, you can’t have this’. ‘No, you can’t be like your friends’. …[with GGC] it was nice to be able to tell them….’Yes, you may have a special birthday dinner’….it was nice to have…that win for the kids and for us as well’*.

Many recipients reported that despite being able to afford more food with the use of GGC, they still needed to employ money-saving strategies to procure sufficient food for their households. These strategies included using price matching programmes at grocery stores or driving to a neighbouring town with the same grocery stores to use GGC because they carried a larger selection of discounted foods. Given that many recipients were still concerned about their ability to afford adequate food, several suggested increasing the number of GGC they received on each occasion or receiving them more frequently.

#### Flexibility and convenience

Since GGC did not have any purchasing restrictions, recipients described having the flexibility to decide when and how much of their GGC to spend, and what types of foods they purchased during each shopping trip. Recipients compared the flexibility of using GGC to the inflexibility associated with accessing food banks which provided them with little to no choice in when they could access food and what foods they received. However, since recipients could only select GGC from among the GGC agencies requested, recipients suggested having the option to select GGC for grocery stores where they preferred to shop, particularly stores that they perceived had more affordable food prices.

Many recipients perceived that GGC were convenient to pick up from their community agency and use at grocery stores as both establishments were close to their home. Programme deliverers also delivered GGC in person or by mail to recipients with transportation barriers. Recipients contrasted this experience of convenient access and use of GGC with the inconvenience of accessing food banks. Recipients reported needing to schedule appointments to visit food banks and often had to make special travel arrangements to pick up food, such as saving money for a taxi, asking a friend or family member for a ride, or having foods delivered by volunteer organisations that could often be unreliable. For instance, Cindy (single-parent household, three children) described the challenges of accessing food banks: *‘it’s difficult [accessing food banks] because I don’t drive, so I had to call in a different company to come deliver…it’s been very difficult getting them to come in…and so then you’re harassing family and waiting for them to have a day off, or taking a day off of work so they can take you’*.

Recipients described feeling a sense of autonomy due to the flexibility and convenience of using GGC because they could decide for themselves what foods to purchase that best met their households’ needs and preferences. Katie (single-parent household, two children) described feeling a sense of pride as GGC provided her with the freedom to purchase foods that she and her family enjoyed compared to being ‘handed’ food: *‘When you’re just handed things, you’re kind of expected to just take what is given to you…. [using GGC] makes me feel like I’m contributing, and doing something for my kids…when you’re able to go out and [pick out your own food]…it just makes you feel human, makes you feel like, yeah, I just did that myself. I didn’t have somebody do it for me’*.

#### Dignified access to food

Programme recipients and deliverers valued the way in which iCAN prioritised recipients’ dignity compared to other food support programmes. For instance, recipients appreciated that GGC were provided discreetly, such as in a sealed envelope or during a one-on-one visit with agency staff. Participants commonly contrasted these experiences with the stigma associated with accessing food from food banks. For instance, Jamie (programme deliverer), described:*‘[GGC provides recipients with] dignity to go shopping…sometimes there’s stigma going to the food bank. Families feel ashamed….kids are embarrassed…[food bank] bags seem to all be dark grey and everybody knows when the families are coming from the food bank’*. The lack of stigma associated with iCAN’s GGC programme enhanced recipients’ social well-being as recipients shared that accessing GGC fostered a sense of connection with their communities. For example, Hakim (two-parent household, three children), described: *‘And the privacy [at the agency where I received GGC], and those who work there, they are fantastic. It’s like everybody there [is] like a family. You get there, [they] ask this, ask that, your health… it’s like [a] community’.*

### Theme 2: Dietary patterns and food skills

#### Food quantity, quality and food skills

With GGC, recipients reported that they were more likely to have adequate nutritious food for their entire families without needing to sacrifice their own intake. Recipients, such as Saleema (two-parent household, three children) reported being able to use GGC to procure more produce for their households: *‘I use [GGC from iCAN] to buy lots of vegetables and fruits, and not any more junk [food]’*. With increased exposure to fresh fruits and vegetables in the home, recipients shared that their children were more likely to request and consume more of these foods and ate fewer pre-packaged and processed foods.

Programme recipients reported that they had greater access to higher quality foods using GGC compared to receiving poor quality foods from food banks. For example, Anna (two-parent household, four children) described, *‘[Food from food banks] goes bad fast. You don’t have a lot of time to eat it. [With GGC], it’s just nice to have fresh vegetables…to have fresh fruit…fresh meat…I definitely enjoy the [GGC] more’*. Many programme recipients also indicated that they appreciated how GGC allowed them to procure the types and amount of food their household needed instead of receiving an overabundance of one food item from food banks that did not meet their food needs. For instance, Francine (two-parent household, six children) reported that the: *‘…[food bank] gave me like six bags of baby spinach. Well, that’s great. I have a couple recipes I can use that in, but I don’t need six bags of it. So if I had a [GGC], I could go and buy my two bags and that would be all I would use’*.

Furthermore, having GGC allowed recipients to prepare home-cooked meals more often and to involve their children in the process. Cooking as a family became a form of family enrichment and skill building for their children. For instance, Kelsey (one-parent household, one child) shared that: *‘[GGC] were nice, because then we got to buy [ingredients] that are usually more expensive… And we make a whole game out of it. I put [recipes from a cookbook] in a container…and then she picks one’ (Kelsey, one-parent household, 1 child)*.

#### Dietary restrictions and cultural food preferences

Recipients who had children with health-related food needs, such as food intolerances or sensitivities, or recipients from households with cultural food preferences reported that they were able to use GGC to afford food that met these specific dietary restrictions and preferences. These were foods that food banks did not provide and were often too costly to purchase through their own financial means. Immigrant recipients appreciated that GGC allowed them to purchase foods that aligned with their culture which reduced food wastage. For instance, Harish (two-parent household, one child) commented that: *‘Like I said about the food bank…you have to understand that [in my culture, I don’t eat beef and] because they put all [the food] together, I don’t feel safe with eating beef and chicken [placed in the same bag] together…otherwise, 50 to 60 % [of the] food [food banks offer] are great, but…I had to throw out [the] other food, and I feel not nice [throwing out food]…that’s why the GGC [from iCAN] really helps because you can choose what you want to buy’*.

### Theme 3: Programme logistical strengths and limitations

#### Connection with clients

Programme deliverers perceived that each time they provided GGC to recipients was an opportunity to engage recipients in conversation and build stronger rapport with them. Programme deliverers perceived that this connection increased recipients’ trust and confidence in programme deliverers to support them in other areas of need, such as skill building programmes and access to mental health support. Some programme deliverers were also able to connect recipients with opportunities to increase their involvement in the community. As Sandra (programme deliverer) shared, *‘[GGC] connected me with families in the community that I wasn’t connected with before…I also had a lot of conversations…with families [receiving GGC]… from there, we’ve now developed a parenting group in the community’*.

#### Programme deliverer workload

Most programme deliverers reported that facilitating iCAN’s GGC programme reduced their overall workload. With GGC, recipients were less likely to access the agencies’ food pantries, so programme deliverers spent less time creating food hampers. Programme deliverers also referred fewer clients to food banks as a result of the GGC programme. As Carmen (programme deliverer) shared, *‘I feel like everybody’s food referrals for the food bank went up [during the COVID-19 pandemic]…when I started getting the [GGC], I didn’t end up doing as many for those families [who received GGC]’*. This reduction in food bank referrals reduced programme deliverers’ administrative workloads as they typically supported recipients in completing and submitting proof of eligibility paperwork each time recipients accessed food banks. By contrast, iCAN did not require agencies to submit proof of eligibility documentation, such as proof of income, to iCAN. Programme deliverers perceived that not requiring proof of eligibility was an advantage because it allowed households with language and other barriers to access iCAN’s GGC programme. Although a few programme deliverers mentioned that facilitating the GGC programme increased their workload, they readily accepted it because they perceived that GGC more effectively met their clients’ food needs.

#### Differential access to grocery gift cards

A key constraint to facilitating the GGC programme was the lack of concrete guidelines governing the distribution of GGC. Instead, agencies developed their own guidelines pertaining to the number of GGC recipients received on each occasion and the frequency with which they received them. Programme deliverers shared that these guidelines were often based on household size, household need and the agencies’ supply of GGC. However, different agencies defined criteria for household size and need differently. Furthermore, although all agencies were aware that iCAN delivered GGC to agencies on a bi-weekly basis, they did not know how many GGC they would receive each time and for which grocery stores, making it difficult to establish an equitable plan for distributing GGC.

Since GGC distribution guidelines differed across agencies, recipients received GGC at varying frequencies depending on the agency they accessed. For example, recipients who received GGC on an *ad hoc* basis reported that they had to contact programme deliverers each time they required GGC, not knowing if there were GGC available or had to wait until programme deliverers contacted them to let them know that GGC were available to pick up. These recipients described feeling a sense of uncertainty as to how they would manage their household food budget when they did not know whether or when they would receive GGC. For instance, Francine (two-parent household, six children) noted: *‘Well if you knew…[GGC] comes, the 15th of every month, or the first of every month, then you know…this is what I have to work with… Like, if I knew I was getting an extra hundred dollars [in GGC], maybe I’m putting 50 [dollars] more to [my utility bill]’*.

#### Programme awareness

The perceived benefits of iCAN’s GGC programme prompted many programme recipients to tell their friends about the programme. Many recipients reported that if they had not been informed by a friend or their caseworker about the programme, they would not have known about it since it was not advertised. As such, programme recipients suggested advertising the GGC programme so that other households in need of food support would benefit from the programme.

## Discussion

This study explored the experiences and perceived outcomes of iCAN’s GGC programme in Calgary, Alberta, Canada, from the perspectives of programme recipients and programme deliverers. Most programme recipients were at risk of food insecurity, and all reported experiencing financial stress about their ability to afford adequate food for their households. Many parents sacrificed their own intake to ensure their children had enough to eat. These experiences of food insecurity were alleviated by participating in iCAN’s GGC programme. Findings from this study demonstrated that both participant groups perceived iCAN’s GGC programme enhanced recipients’ sense of autonomy and dignity through financial support, the flexibility and convenience of using GGC, and the freedom to select foods they desired. Recipients noted that these benefits helped to improve their social and emotional well-being. Recipients also reported that their households’ dietary patterns improved as GGC allowed them to select and procure fresh, higher quality foods that were not often provided by other charitable food support programmes. Improvements in food skills were also noted as recipients were able to prepare more home-cooked meals. From programme deliverers’ perspectives, facilitating iCAN’s GGC programme strengthened their rapport with recipients, offering them opportunities to increase recipients’ social connections to the community. Both participant groups also highlighted programmatic strengths, including that iCAN did not require agencies to submit complex eligibility documentation to iCAN which contributed, in part, to a reduction in programme deliverers’ overall workload. Study findings also pointed to some limitations of iCAN’s GGC programme, including a lack of concrete guidelines governing GGC distribution to agencies and differing GGC timing and distribution guidelines to recipients across agencies. Nonetheless, all recipients preferred to access food support from iCAN’s GGC programme rather than from food banks.

iCAN’s GGC programme enhanced recipients’ access to food by addressing all five domains of Freedman et al’s^(33)^ framework, particularly the economic domain. Within the economic domain, both participant groups perceived that GGC increased household financial resources, which all participants perceived was a key advantage of iCAN’s GGC programme as it alleviated recipients’ stress over their finances and food supply. Reducing parents’ stress related to household food insecurity is important for children’s well-being as studies have demonstrated that children from food-insecure households are aware of and take on parents’ stress related to household food insecurity^(35,36)^. Studies have also shown that children from food-insecure households experience feelings of worry, anxiety and stress in response to constrained household finances and food supply^(36,37)^.

In the spatial-temporal domain, programme recipients and deliverers indicated that they preferred the close proximity of community agencies and grocery stores to their homes to pick up and use GGC compared to the inconvenience of accessing food banks. Other studies have similarly found that participants reported difficulties accessing food banks due to transportation barriers and scheduling conflicts, which were primary reasons food-insecure households stopped accessing food banks despite their need for food^(38–40)^.

In the service delivery domain, recipients described that the flexibility and convenience of accessing GGC from agency staff and using GGC at grocery stores were notable benefits of iCAN’s GGC programme. Food subsidies that allow unrestricted food purchases, such as iCAN’s GGC programme or the SNAP in the USA, have been a controversial topic in the literature as there are concerns that unrestricted subsidies may not improve diet quality, since households can still purchase less nutritious foods (e.g. soda or candy)^(24,41,42)^. However, our findings demonstrated that the lack of purchasing restrictions allowed recipients to select and procure more fresh fruits and vegetables and foods that aligned with their household’s cultural preferences and food needs. This sense of autonomy over their food choices allowed recipients to plan and prepare meals at home more often as a family. Involving children in family meal preparation is associated with improved dietary patterns and self-efficacy, which may reduce children’s risk of food insecurity in adulthood^(43,44)^. Recipients commented that they did not have this sense of autonomy when accessing food banks, which is consistent with previous studies of food bank users in which they perceived having little to no control over the types and quality of foods they received^(38,39)^. Furthermore, iCAN did not require recipients to provide proof of eligibility to receive GGC because agency partners were already aware of which households were most in need. Prior studies have shown that food subsidy programmes with stringent eligibility requirements (e.g. income thresholds) and complex enrolment processes were barriers to participation for many food-insecure households^(25,45)^. Thus, iCAN’s programme delivery model may have facilitated access to food for a greater number of food-insecure households who may have otherwise had to go without, including households with language barriers.

The lack of stigma and shame associated with accessing iCAN’s GGC programme and using GGC enhanced recipients’ access to food in the social domain. These findings are consistent with other studies, which found that participants consistently viewed food subsidies as a less stigmatising and more dignified approach to accessing food support than food banks^(20,23)^. The use of food banks has been explicitly linked with feelings of shame and failure among parents in their inability to provide sufficient, nutritious food for their children^(46,47)^. By contrast, recipients reported feeling a sense of pride and agency using GGC as GGC allowed them to support not only their children’s food needs but also to provide their children with ‘special moments’, such as being able to celebrate children’s birthdays. iCAN’s GGC programme also created opportunities for programme deliverers to strengthen their rapport with recipients, which supported recipients in feeling less isolated and better connected to their community.

In the personal domain, participants from both groups reported that GGC provided recipients with greater access to foods that met their household’s health-related dietary needs which were rarely available from food banks. By contrast, food banks are often unable to meet recipients’ health-related dietary needs due to their reliance on food donations from the public and food retailers^(22,38,39)^.

In addition to the positive experiences and outcomes of iCAN’s GGC programme, recipients and programme deliverers also identified programmatic constraints. Many recipients still relied on money-saving strategies to afford sufficient food for their households. In addition, some recipients received GGC consistently, while others received them on an *ad hoc* basis. Those who received GGC on an *ad hoc* basis reported that this approach created a sense of uncertainty and limited their ability to effectively manage their household food budget and food supply.

As household food insecurity rates continue to rise in Canada^(3)^, GGC programmes may help to ensure households have access to adequate and nutritious foods. Findings from this study were used to inform three recommendations that may further enhance the programme’s feasibility and possibly augment the amount of support iCAN could provide to households at risk of food insecurity in the future. Notably, iCAN has now implemented these recommendations. First, as food prices are expected to continue to rise^(48)^, it would be helpful to increase the number of GGC provided to recipients on each occasion, particularly for those living in larger households and during the summer months when school meal programmes are not accessible. Second, iCAN should attempt to provide clear expectations regarding the number of GGC agencies can expect to receive at one time and for which stores and should establish consistent criteria pertaining to how often and how many GGC recipients can receive. Third, increasing awareness of iCAN’s GGC programme may help to increase donor contributions to the programme. iCAN is a non-profit organisation that strives to improve access to food for food-insecure households with children under 18 years of age. The sustainability of iCAN’s GGC programme and its continued reach to such households depends on monetary donations from corporate and private donors. As such, an increase in donor contributions is essential if iCAN is to provide more certainty to programme recipients and deliverers regarding the number and frequency with which they can expect to receive GGC. An increase in donations would also increase iCAN’s ability to support more households at risk of food insecurity. However, it is not clear whether it is reasonable to expect monetary donations to increase given current economic conditions (e.g. high inflation).

Despite this study’s strengths, there were also several limitations. Although all participants spoke English, several participants were not entirely fluent, which may have impacted how well they understood and responded to interview questions and, at times, how well the interviewers understood their responses. Selection bias was also possible as some populations at high risk of food insecurity were not interviewed, such as households experiencing homelessness or with a parent/caregiver living with a disability. These exclusions may have limited the breadth of experiences reported by participants. Study findings are also specific to participants who accessed and delivered iCAN’s GGC programme. Findings, therefore, may not be generalisable to the experiences and perceived outcomes of recipients and programme deliverers of GGC programmes in other regions of Canada or other countries. Future research should include quantitative studies to understand the long-term implications of receiving GGC on household food insecurity risk, diet quality, and physical and mental health.

### Conclusion

To our knowledge, this is the first qualitative study to explore the experiences and perceived outcomes of a GGC programme. Programme recipients and deliverers reported that iCAN’s GGC programme increased recipients’ access to nutritious food in a way that enhanced their sense of autonomy and dignity and improved their households’ finances and dietary patterns. They also reported that accessing iCAN’s GGC programme enhanced recipients’ social and emotional well-being. Programme deliverers perceived that facilitating iCAN’s GGC programme allowed them to strengthen their connections with their clients and reduced their overall administrative workload. The latter may enhance the feasibility of delivering GGC at scale. Increasing the number of GGC provided to households on each occasion, setting clear guidelines for distributing GGC to recipients and increasing potential donors’ awareness of iCAN’s GGC programme may increase the amount of support iCAN could provide to households at risk of food insecurity in the future. However, it is important to remember that the causes of household food insecurity are multifactorial, and therefore no single programme or policy can resolve it. A whole-of-society approach is needed in which all sectors – including governments, organisations and citizens – collaborate to ensure that individuals and households have adequate income to purchase sufficient food and other basic necessities. In this way, GGC and other food subsidy programmes can be one important piece of a comprehensive approach to address household food insecurity in Canada.

## Supplementary material

For supplementary material accompanying this paper visit https://doi.org/10.1017/S136898002300157X. click here to view supplementary material
