# Supplementary material for: Experiences and perceived outcomes of a grocery gift card programme for households at risk of food insecurity
Source: Public Health Nutr. 2023 Aug 2;26(11):2460–9. doi: 10.1017/S136898002300157X (PMC10641610; doi:10.1017/S136898002300157X)
Supplement: Supplementary file 1 [file S136898002300157Xsup001.docx]

**Supplementary Material**

**Data Supplement 1: Program recipient interview questions and probes**

| **Experience accessing iCAN’s GGC program** |
| --- |
| - What was it like to get grocery gift cards from I Can for Kids?   *Sample probes:*   - Where could you use the grocery gift cards? - What did you not like or like about getting and using grocery gift cards from I Can for Kids? - What made it hard or easy to pick up or use grocery gift cards? |
| **Perceived outcomes of accessing iCAN’s GGC program:** |
| - How has getting grocery gift cards from I Can for Kids impacted you? Your children? Other household members?   *Sample probes:*   - How has it impacted you negatively? - How has it impacted you positively?   *Probes inquired each of Freedman’s five domains (able to purchase foods that aligned with culture and/or health-related dietary needs, transportation to grocery stores to use grocery gift cards, impact on household finances, physical/mental/social well-being)*   - What would it be like if your household did not get grocery gift cards from I Can for Kids? - What could be done to make grocery gift cards from I Can for Kids better? - What would you change about grocery gift cards from I Can for Kids to better meet your child(ren)’s needs for food? What would you keep the same? |
| **Experience accessing other food support programs:** |
| - Have you gotten food hampers before? For example, from I Can for Kids or the Food Bank. Yes/No - If **no**: move on to next question - If **yes**: What was the experience of getting a food hamper like for you and your household? What did you like or not like about food hampers? How does that compare to grocery gift cards?   *Probes inquired each of Freedman et al’s five domains (e.g. transportation to food banks, how foods fit with cultural preferences, food quality, impact on household finances, physical/mental/social health)* |

**Data Supplement 2: Program deliverer interview questions and probes**

| **Experience facilitating iCAN’s GGC program** |
| --- |
| - What feedback have you received from households about grocery gift cards from I Can for Kids?   *Sample probes:*   - When did households start receiving grocery gift cards from I Can for Kids? - Based on feedback you have received from households, what do households like about receiving grocery gift cards from I Can for Kids? What did they not like? - What makes it easy or hard for households to pick up grocery gift cards? - What is your experience of distributing grocery gift cards from I Can for Kids?   *Sample probes:*   - Tell me about the process of distributing grocery gift cards to households. - How do you decide how many grocery gift cards to provide households? - How do you decide how often to distribute grocery gift cards to households? - What parts of distributing grocery gift cards do/do not work well? |
| **Perceived outcomes of facilitating iCAN’s GGC program** |
| - How have grocery gift cards from I Can for Kids impacted children and their household?   *Sample probes:*   - How have they impacted children and their households negatively? - How have they impacted children and their households positively?   *Probes inquired each of Freedman’s five domains (able to purchase foods that aligned with culture and/or health-related dietary needs, transportation to grocery stores to use grocery gift cards, impact on household finances, physical/mental/social well-being)*   - What would it be like if households did not get grocery gift cards from I Can for Kids? - How has distributing grocery gift cards from I Can for Kids impacted you?   *Sample probes:*   - How did facilitating I Can for Kid’s grocery gift card program impact your workload? - How did facilitating the program impact your connection with clients? - What could be done to make grocery gift cards from I Can for Kids better? - What would you change about grocery gift cards from I Can for Kids to better meet children’s needs for food? What would you keep the same? |
| **Perceived household experiences of other food support programs** |
| - How do you think that households’ experiences and impacts of receiving grocery gift cards compare to receiving a food hamper?   *Probes inquired each of Freedman et al’s five domains (e.g. transportation to food banks, how foods fit with cultural preferences, food quality, impact on household finances, physical/mental/social health)* |
